# Near-Complete Genome Sequence of Ndumu Virus from Garissa, Kenya, 1997

**DOI:** 10.1128/MRA.00551-21

**Published:** 2021-08-26

**Authors:** Naazneen Moolla, Natalie Viljoen, Venessa Patharoo, Antoinette Grobbelaar, Arshad Ismail, Jacqueline Weyer

**Affiliations:** a Centre for Emerging Zoonotic and Parasitic Diseases, National Institute for Communicable Diseases, National Health Laboratory Service, Johannesburg, South Africa; b Department of Medical Virology, Faculty of Health Sciences, University of Pretoria, Tshwane, South Africa; c Department of Microbiology and Infectious Diseases, Faculty of Health Sciences, University of Witwatersrand, Johannesburg, South Africa; d Division of Virology, Faculty of Health Sciences, University of the Free State, Bloemfontein, South Africa; e Sequencing Core Facility, National Institute for Communicable Diseases, National Health Laboratory Service, Johannesburg, South Africa; Queens College CUNY

## Abstract

We report a nearly complete genome sequence of Ndumu virus (NDUV) identified using a metagenomics approach. The sequence was derived from a viral isolate obtained from a bovine calf following a diagnostic investigation of the 1997 to 1998 Rift Valley fever (RVF) outbreak in the Garissa District of northeastern Kenya.

## ANNOUNCEMENT

Isolated from Mansonia uniformis mosquitoes in South Africa in 1959, Ndumu virus (NDUV) belongs to the family *Togaviridae*, genus *Alphavirus* (1, 2). NDUV has also been detected in mosquitoes in Kenya (3, 4) and Senegal (5). NDUV was identified in domestic pigs and the blood meals of mosquitoes that fed on goats and sheep in a study conducted in Uganda (6, 7). The pathogenicity of the virus in humans remains unclear, although serological evidence of exposure in humans exists (1).

A blood sample collected from a calf presenting with Rift Valley fever (RVF)-like symptoms during the RVF outbreak in Kenya was submitted for diagnostic investigation to the National Institute for Communicable Diseases (NICD) (then the National Institute for Virology), South Africa (8). The sample tested negative for RVF (8), but an unidentified virus isolate was obtained during the investigation by passage in suckling mouse brains and amplified by serial passage in Vero cells (ATCC CRL-1586). The cleared lysates from infected Vero cells were stored frozen. The identity of the latter remained unknown until the investigation reported here. The isolate was retrieved from the archival collection of the Centre for Emerging Zoonotic and Parasitic Diseases, NICD, and was passaged once on Vero C1008 cells (ATCC CRL-1586) using standard cell culture techniques. Viral genomic RNA was extracted from the culture supernatant using the QIAamp viral RNA kit (Qiagen, Germany), and sequence-independent single-primer amplification (SISPA) as detailed by Djikeng et al. was performed after DNase treatment (9, 10). Briefly, cDNA was retrotranscribed from the extracted RNA using the SuperScript III kit (Invitrogen, USA) and the primer FR-26-RV-N (9). Second-strand DNA was synthesized using Klenow exo-DNA polymerase. Purified cDNA was amplified using SISPA with MyTaq Red mix (Meridian Bioscience USA), using the primer FR-20-RV (9). The DNA was quantified using a Qubit fluorimeter (Thermo Fisher Scientific, USA); paired-end libraries were prepared using the Nextera DNA Flex library preparation kit, followed by sequencing (2 × 150 bp) on a NextSeq 550 platform (Illumina, Inc., USA). A total of 4.99 million sequencing reads were obtained with an average read length of 149 bp. The FASTQ files were uploaded to the Galaxy Web platform, and the data were analyzed using the public server at http://usegalaxy.org/ (11). All tools were run for paired-end reads using default parameters unless otherwise specified. The reads were quality trimmed (qualified quality Phred score, 20; length required, 40) using fastp V0.20.1 (12) and classified using Kraken V1.1.1 with the viruses database (13); 1.72 million reads were mapped to NDUV. The paired-end reads were mapped against the NDUV (GenBank accession number NC_016959.1) reference sequence using Bowtie2 V2.3.4.1 with the very sensitive end-to-end preset (14). The mapped reads were curated into a pileup file using SAMtools V1.9 (15), the consensus sequence determined using iVar V1.2.2 (16), and annotation performed using Vgas (17) at http://guolab.whu.edu.cn/vgas. The sequence was 11,648 nucleotides long with 50.1% GC content and an average sequencing coverage of 7,612×. The sequence obtained had 98% nucleotide and 99% amino acid similarity to the reference sequence as determined using Sequence Identity and Similarity (SIAS), available at http://imed.med.ucm.es/Tools/sias.html. Preliminary analysis suggested that three nucleotide insertions (NC_016959.1:g.7427_7428insGC; NC_016959.1:g.7440_7441insG) may affect the coding sequence of the structural polypeptide [YP_005351233.1:p.(Pro13_Pro17delinsArgGlnProTyrArgArg) ins(1)]. The identification of NDUV from bovines has not been previously reported, to our knowledge. These data also contribute to the limited genomic sequence data available for NDUV.

### Data availability.

This sequence has been deposited in GenBank under the accession number MZ091531. The version described here is the first version. The raw reads were deposited in the NCBI Sequence Read Archive (SRA) under the accession numbers PRJNA728540 (BioProject) and SAMN19091165 (BioSample).
